# Implementation of Blockchain Technology Could Increase Equity and Transparency in Organ Transplantation: A Narrative Review of an Emergent Tool

**DOI:** 10.3389/ti.2023.10800

**Published:** 2023-02-08

**Authors:** Alessandro Anselmo, Marco Materazzo, Nicola Di Lorenzo, Bruno Sensi, Camilla Riccetti, Maria Teresa Lonardo, Marco Pellicciaro, Francesco D’Amico, Leandro Siragusa, Giuseppe Tisone

**Affiliations:** ^1^ Department of Surgical Science, University of Rome “Tor Vergata”, Rome, Italy; ^2^ Transplantation and Hepatobiliary Surgery, University of Padova, Padova, Italy

**Keywords:** transplants, blockchain, delivery of health care, electronic health records, transplantation conditioning, social justice, equality

## Abstract

In the last few years, innovative technology and health care digitalization played a major role in all medical fields and a great effort worldwide to manage this large amount of data, in terms of security and digital privacy has been made by different national health systems. Blockchain technology, a peer-to-peer distributed database without centralized authority, initially applied to Bitcoin protocol, soon gained popularity, thanks to its distributed immutable nature in several non-medical fields. Therefore, the aim of the present review (PROSPERO N° CRD42022316661) is to establish a putative future role of blockchain and distribution ledger technology (DLT) in the organ transplantation field and its role to overcome inequalities. Preoperative assessment of the deceased donor, supranational crossover programs with the international waitlist databases, and reduction of black-market donations and counterfeit drugs are some of the possible applications of DLT, thanks to its distributed, efficient, secure, trackable, and immutable nature to reduce inequalities and discrimination.

## Introduction

Since the first kidney transplantation in 1957, transplantation emerged as a novel exciting discipline focused on innovative encompassing drug design, translational medicine, surgery, and ethics (1).

Besides medical innovation, in the last few years, innovative technology and healthcare digitalization played a major role in all medical fields. For instance, electronic medical records (EMR) changed daily practice, providing the future chance for big data analysis and artificial intelligence application (2). To ease this digital revolution, a great effort has to be made by the national health system (NHS) to manage this large amount of sensitive data, paying maximum attention to security and digital privacy, a novel human right recognized by the United Nations (3). Among all patients, the security and digital privacy of the people on the transplant waiting list and also post-transplantation is even more urgent due to the amount and nature of the data (e.g., donor data). Moreover, this cohort of patients represent a population small enough to safely evaluate the application of novel technology in clinical care.

**FIGURE 1:**
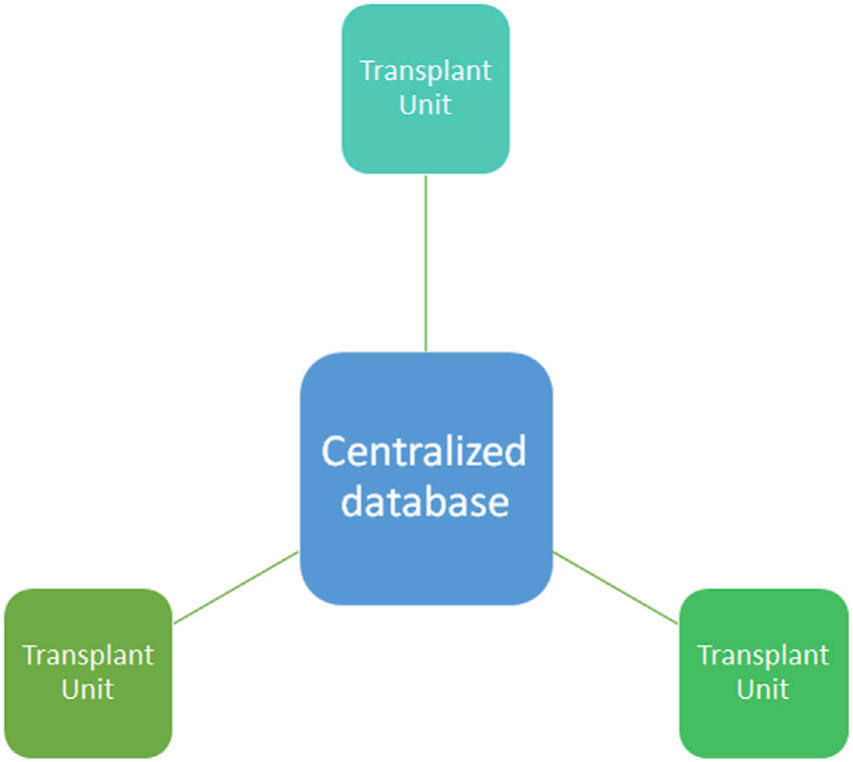
Centralized database system. In a centralized database system all the information are stored in the centralized database where the different transplant units (nodes) upload the data from donors, recipients, and clinical outcome. Centralized database are more prone than other technology to cyberattacks and hacking.

In light of this, a great effort has been made by the local transplant program coordinator to design a transparent and fair organ allocation system and to overcome illegal practice (4) with a centralized system and centralized data storage (Figure 1). Despite cryptography, centralized database systems are more prone to cyberattacks and hacking, like the last ransomware attack on COVID-19 vaccination registration portal in 2021 in Lazio (an Italian region) (5). Moreover, COVID-19 itself determined a further decentralization and increase in telehealth assessment (6–11). In order to solve some of these issues, blockchain technology has been proposed as a possible solution by several authors (12).

Blockchain technology, designed by a mysterious author named Satoshi Nakamoto in 2008, defines a peer-to-peer distributed database without centralized authority (3). Initially applied to the Bitcoin protocol, blockchain technology offered trustable decentralized electronic cash transactions without any validation from trustable third party (TTP) institutions.

Specifically, a blockchain is a record of a peer-to-peer transaction made by linked transaction blocks that are immutable and shared in a network. Every node of the network has a copy of the distribution ledger, defined as “a type of database which is shared, replicated, and synchronized among members of a network. The distribution ledger records the transaction, such as assets or data, among the participants of the network.”

Blockchain can be classified according to the accessibility of the distribution ledger as public, private, and permissioned blockchains. A public blockchain is anonymous and any user can have a copy and participate in confirming a transaction, whereas, in a private blockchain, the distribution ledger is controlled by the owner who regulates all the aspects of the network and can even change the content of the blockchain itself. Permissioned blockchain represents an intermediate solution where an organization supervises the admission of the individuals, the allocation of the distribution ledger to individuals, and the permission to confirm transactions.

**FIGURE 2:**
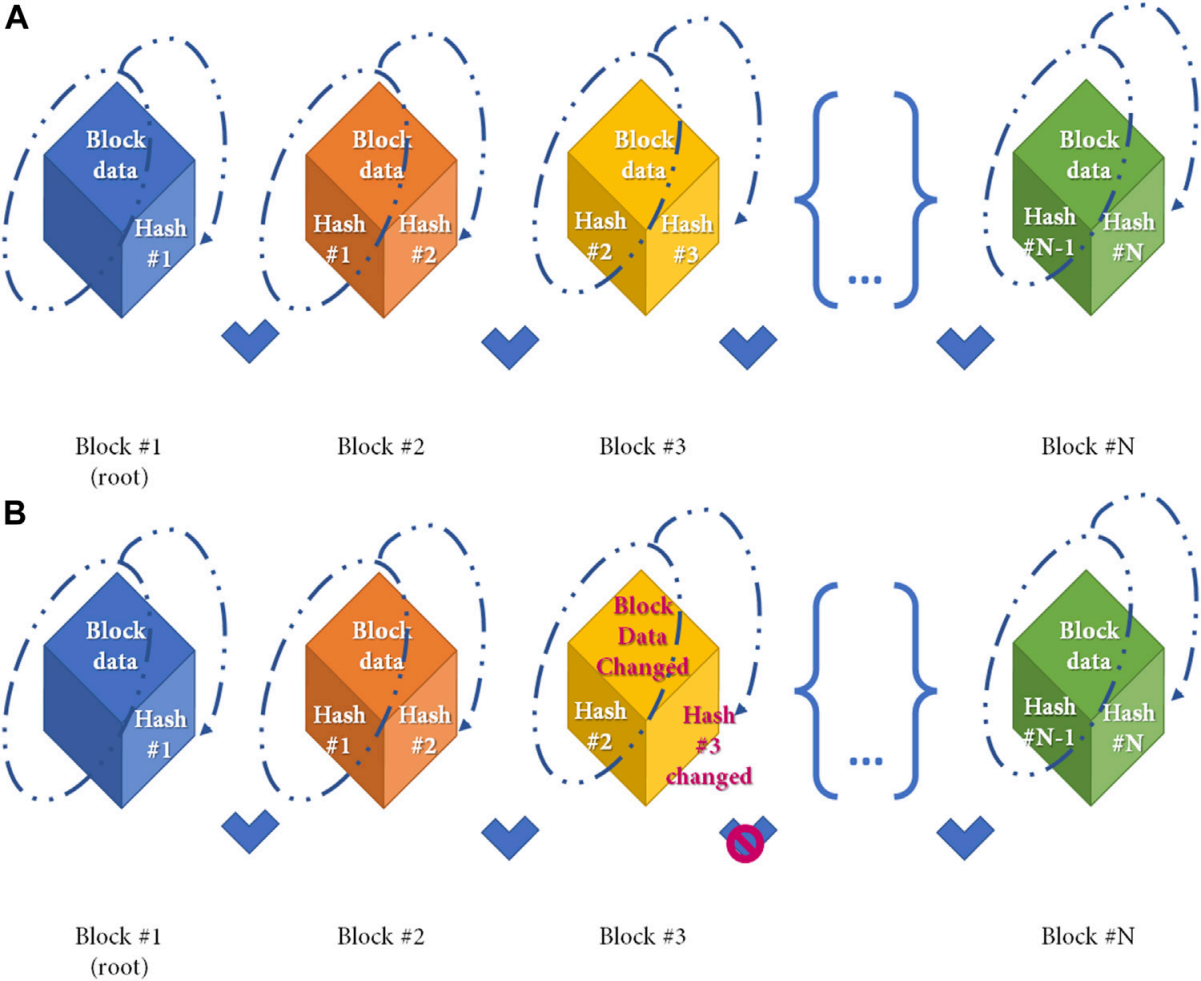
Simple Blockchain model. Distribution ledger technology (DLT) is a type of database which is shared, replicated, and synchronized among members of a network. DLT is made by single different blocks which contains the data recorded in the DLT. **(A)** Simple Blockchain model. Every cube represents a different block. Any single block after the first is made by 2 different hashes and the data, as shown in the figure. A hash function is any function that can be used to map data of arbitrary size to fixed-size values and is produced during the Proof-of-Work to ensure sequentality of DLT. Any block from the DLT contains the hash from the previous block and the hash of itself. Block hash is calculated by the data contained in the block, and the previous hash (as shown in dotted line) **(B)** If the data contained in the block is changed (e.g., Block#3) the block hash (hash#3) will change with a denial of the block based on the incorrect association between blocks.

**FIGURE 3:**
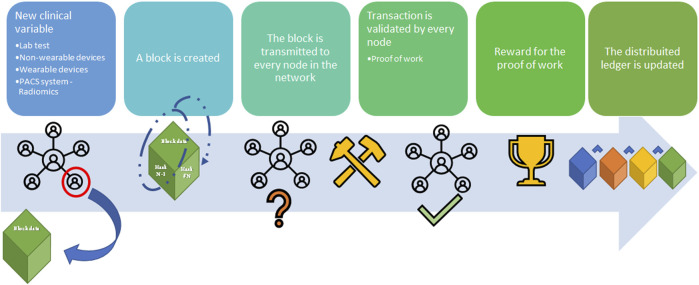
Blockchain workflow sample (from left to right). A node in the blockchain submits the request to create a new block with an amount of data (supply chain; cryptocurrency transaction; medical record), namely Block (n). Block (n) is made by the node to contain the hash from Block (n–1), hash (n–1), and the data. Block (n) is transmitted to every node of the network without hash (n). Hash (n) for Block (n) is calculated from the data in the block [hash (n-1) plus the data] in every single node through the Proof-of-Work (PoW) (so-called mining) to avoid malicious entities. Every node validates the new block Every node receives the reward for the PoW and the Block (n) is added to the blockchain.

Figure 2 shows a simple distribution ledger (blockchain) made by N blocks. Every single block contains data (N) (e.g., money transactions, supply chain data, medical data, etc.) with timestamps, a hash of the previous block (n–1), and a hash of what is contained in the block (hash n–1 plus data of n). The security of this protocol lies in the hash that links one block to the next one. If any data is changed in the block, then the hash created for the block and the next one will be incorrect. Due to the distributed nature of the blockchain, if any data modification is made, any node that has a copy of the chain should modify accordingly to maintain coherence in the sequence, a highly unlikely situation in the public and permissioned blockchain (high Byzantine fault tolerance). Finally, the sequence is secured by another mechanism: Proof-of-work (POW) consensus. PoW consensus represents a time-consuming mathematical function that is required prior to validation of the block as a deterrent for malicious access. After blockchain spread several different alternative to PoW have been designed to reduce energy and/or time consumption (13). Figure 3 shows all the workflow required to add a block to the distributed ledger.

Beyond the birth of several cryptocurrencies emulating the Bitcoin experience (14), several non-medical industries started to apply the blockchain technology in several fields, thanks to the sustainability, and the lack of central agency in several supply chains in perishable goods, such as fish, or non-perishable goods such as diamonds (15). Regarding medical data, the promising experience of the Estonian NHS to secure EMR with blockchain technology demonstrates its technical applicability in medical fields.

Taking into account these non-medical and medical experiences, the aim of the present review is to establish the future role of blockchain and distribution ledger technology (DLT) in the organ transplantation field. In order to help transplant physicians to familiarize the DLT technology. Table 1 summarizes some of the non-medical vocabulary used.

**TABLE 1:** Non-medical vocabulary used in review.

**Blockchain:** Blockchain is a record of a peer-to-peer transaction made by linked transaction blocks that are immutable and shared in a network.
**Distribution ledger:** a type of database which is shared, replicated, and synchronized among members of a network. The distribution ledger records the transaction, such as assets or data, among the participants of the network.
**Hash:** A hash function is any function that can be used to map data of arbitrary size to fixed-size values. In the blockchain technology is used to ensure the sequentiality of the data in the blockchain.
**Internet of thing (IoT) technology:** a network of physical things linked to each other by means of the Internet (16),
**Machine-to-machine communication (M2M):** a particular system network where machines communicate without human involvement, avoiding human manipulation and securing organ allocation system (18).
**Non-fungible Token (NFT):** is a unique digital identifier that cannot be copied, substituted, or subdivided, that is recorded in a blockchain, and that is used to certify authenticity and ownership.
**Proof-of-work (PoW) consensus:** a time-consuming mathematical function that is required prior to validation of the block as a deterrent for malicious access. Figure 3 shows all the workflow required to add a block to the distributed ledger.
**Ransomware:** is a type of malware from cryptovirology that threatens to publish the victim’s personal data or permanently block access to it unless a ransom is paid.
**Trustable third party (TTP):** an entity which facilitates interactions between two parties who both trust the third party; the Third Party reviews all critical transaction communications between the parties, based on the ease of creating fraudulent digital content.

## Materials and Methods

### Search Strategy

A systematic review was designed to analyze all the early experiences of DLT in trasplantology and was conducted according to the Preferred Reporting Items for Systematic Reviews and Meta-Analyses (PRISMA) guidelines (16). The protocol of this systematic review was registered in PROSPERO (CRD42022316661). A systematic literature search of Medline, Web of Science, and Cochrane databases with the following search string: (“blockchain” or “distributed ledger technology”) AND (“transplant” OR “graft”) was carried out on 29th April of 2022, and additional manuscripts were retrieved from reference lists of included studies and relevant reviews. Moreover, a grey literature search was performed through Google to find other resources available.

Results have been imported in Mendeley 1.19.8 (Elsevier, Netherlands) to remove duplicates. Bibliographic citations of included studies have been manually searched to identify other studies that filled the review’s inclusion criteria.

### Selection of Studies

Two reviewers (MM and MP) worked independently to screen the titles and abstracts of identified citations, and subsequently, the full texts of potentially eligible studies. Disagreements between reviewers were resolved by discussion and with the help of a senior adjudicator (AA).

### Data

Eligible studies were all English manuscripts regarding DLT application in organ and tissue transplant, even partially. Manuscripts regarding other specialties were excluded from the study.

## Results

### Literature Search and Study Characteristics

**FIGURE 4:**
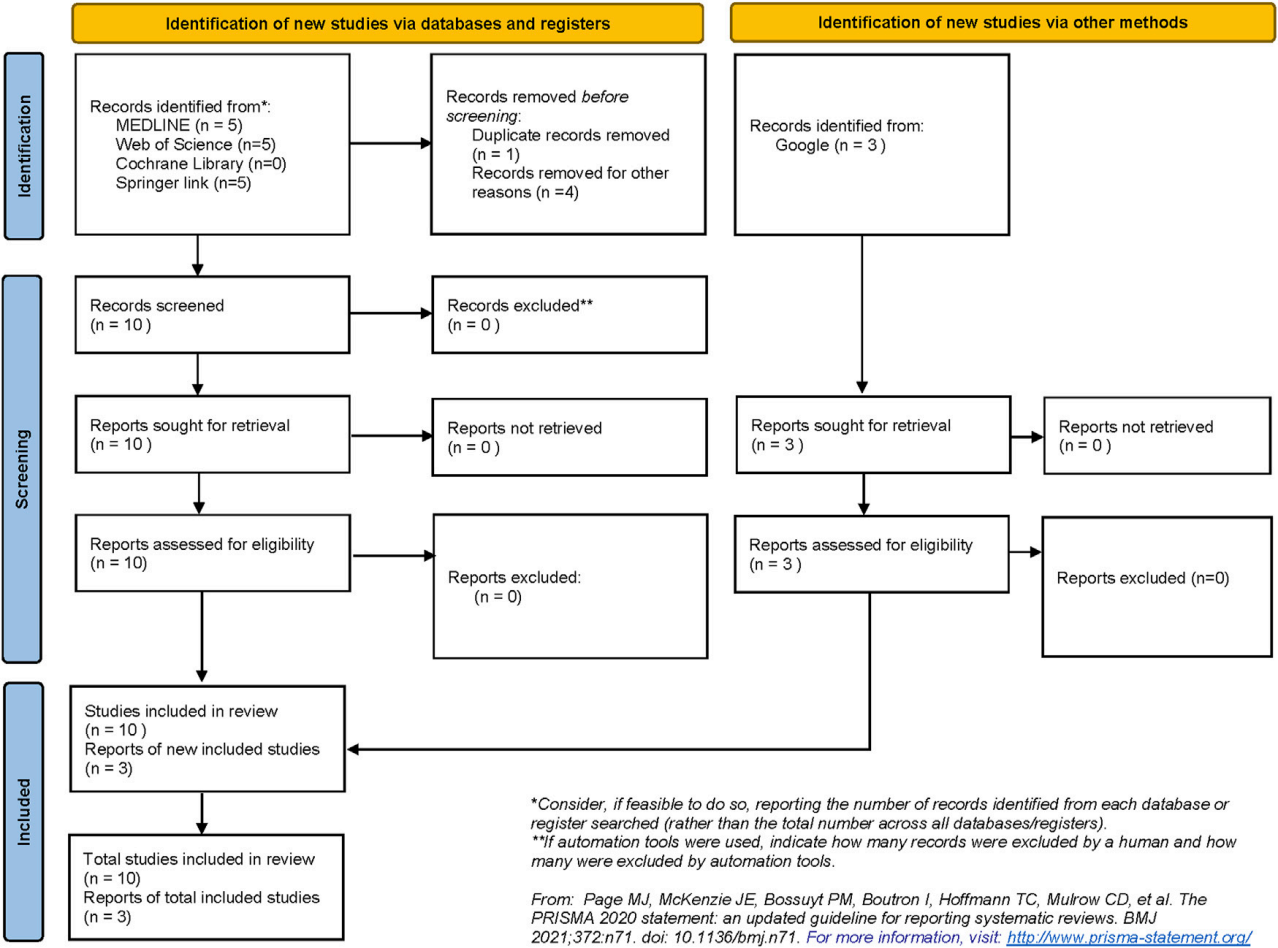
Preferred Reporting Items of Systematic Reviews and Meta-Analyses (PRISMA) flow diagram demonstrating the process of study selection in the review.

The systematic search strategy identified a total of 13 publications that were included in the narrative review (Figure 4). None of the publications enlisted were registered as clinical trials. Therefore, due to the lack of a clinical outcome and paucity of data, a narrative review was designed to underline both already designed and putative future applications of DLT in transplant care.

### Preoperative Assessment

#### Deceased Donor Organ Allocation, DLT, and the Internet of Thing (IoT)

Organ allocation systems encompass all the processes involved in organ distribution across a region to ensure fair and ethical distribution across patients on the waiting lists (17–19). Currently, to our knowledge, at least six different models of DLT for organ allocation were described in the literature by Jain, Ranjan et al., Dajim et al., Lamba et al., Alandjani, and Daniel et al., (14, 20–24)*.* In their manuscript, Jain focused his work on different DLT models (OrganChain) for organ allocation namely Scheme A (Matching organ inside the Blockchain) and Scheme B (Matching organ inside the Blockchain), with Scheme A resulting in superior generating fewer blocks (22). To evaluate the performance of their blockchain-based system, four variables were evaluated: maxed batch time out, max block size, endorsement policy, and transaction rate (25).

Another peer-to-peer and secure protocol network was presented by Ranjan et Al. and called Interplanetary File System (IPFS). IPFS aimed at reducing the price of uploading donors’ and patients’ EMRs. DLT presented was secured with double hashing (14).

Notably, Dajim et al. focused their work on overwhelming the issue of their current donation and transplantation system in Saudi Arabia (lack of transparency, data security, and privacy) (23). Prevention of black-marketing issues in organ donation and transplantation was the main objective of the model provided by Lamba et al. (24)*.*

The other studies published by Alandjani, and Daniel et al., focused their work on the development of DLT and their evaluation on IoT, consumption, scalability, and gas consumption (20, 21). All the distributed allocation systems are designed on Hyperledger fabric, a Linux Foundation open source project (26), or Ethereum, a decentralized, open-source blockchain with smart contract functionality. DLT applications in organ allocation are summarized in Table 2.

**TABLE 2:** Summary of the Organ allocation system developed.

Authors	DLT used	Contributions
Jain (22)	Hyperledger Fabric	OrganChain prototype to discover the performance of a blockchain-based OPTN
Ranjan et al. (14)	Ethereum	InterPlanetary File System (IPFS) to Reduce the cost to upload donor and patient data
		Double hashing technique for proving security and privacy for donor’s and patient’s data
Dajim et al. (23)	Ethereum	Overcoming the limitations of Saudi Arabia’s transplantation system (lack of transparency, data security, and privacy)
Lamba et al. (24)	Hyperledger Fabric and Hyperledger Composer	Prevention of organ black-market
Alandjani (21)	—	Scalability
		IoT application in DLT technology
Daniel et al. (20)	Ethereum	Scalability and gas consumption

DLT, distribution ledger technology; OPTN, Organ procurement transplant network; IoT, Internet-of-Thing.

## Discussion

### Deceased Donor Organ Allocation, DLT, and the Internet of Thing (IoT)

Organ allocation systems encompass all the processes involved in organ distribution across a region to ensure fair and ethical distribution across patients on the waiting lists (18, 19). The importance of ethical organ allocation lies in the huge number of patients in the waiting list; it has been calculated that every 12 min a new name is added to the organ waiting lists and that an average of 21 patients die due to lack of organ availability every day (21). Due to the increasing demand for organs and the inadequacy of organ procurement, every country designs its own allocation rules trying to balance inequality among patients (utility model) and transplant benefit (net life-years gained) (19).

For instance, in Italy, deceased kidney donor allocation includes a regional level where several factors such as waiting time, age, human leukocyte antigen (HLA) match, % of panel reactive antibody (PRA), defined regional-based or national-based renal urgency, combined transplant, and pediatric priority are taken into consideration (18).

Another example of deceased organ allocation is the model for deceased liver distribution among countries in the Eurotransplant program. In the Eurotransplant model, liver donors are allocated first internationally to high urgency status patients or to those with an approved combined organ status, and then on a national basis, where allocation is recipient-driven or center-driven, depending on local rules (27). In the latter case, “match MELD,” AB0 blood group rules, predefined center, and donor profile criteria (age, weight, virology, split, etc.) for a particular recipient, and time from the listing are all taken into account prior to organ offering (27). “Match MELD” consists of the highest value between “lab MELD” or “exceptional MELD.” “Lab MELD” is calculated according to the Model for End-Stage Liver Disease (MELD) (28) with international normalized ratio (INR), bilirubin level, and serum creatinine. The latter, exceptional MELD, can be requested under certain circumstances when patient severity is not well described by lab MELD with the disease list repeatedly revised (27).

However, despite the wide application of complex allocation system, in some countries, the lack of a connecting platform could ease illegal practices or illegitimate methods in some hospitals (14). It has been calculated that 5%–10% of kidney transplants performed annually are currently through illegal practices, such as organ trade, and organ tourism (29). Moreover, illegal organ donation lacks all the preoperative assessment of recipient and donor to reduce possible side effects, oncological and infective risks (30, 31). Under these circumstances, fair organ allocation is an ethically compelling need in order to prevent harm to patients and on transplant program reliability worldwide.

Hence, DLT technology could provide a useful tool to resolve these issues, providing an efficient, secure, distributed, trackable, and immutable framework to promote organ allocation and donation (14, 21). Firstly, the DLT model through decentralization and without a centralized source could determine a sharing model to cope with such security threats and anonymity of data transactions (21, 22). A possible model for DLT in organ allocation and listing may be represented by a permissioned blockchain network where regulatory authority can easily control the access in the network. Permissioned blockchain rely on a governance structure (in this case regulatory authority) that controls access and enforces rules. In this specific blockchain network commonly are implemented alternative computationally intensive consensus mechanisms compared to PoW, because of the degree of trust among the different nodes. In a permissioned DLT, regulatory authority, as in centralized network, are in charge of responding to incident including cyber threats and as in a centralized network they can control access (13).

**FIGURE 5:**
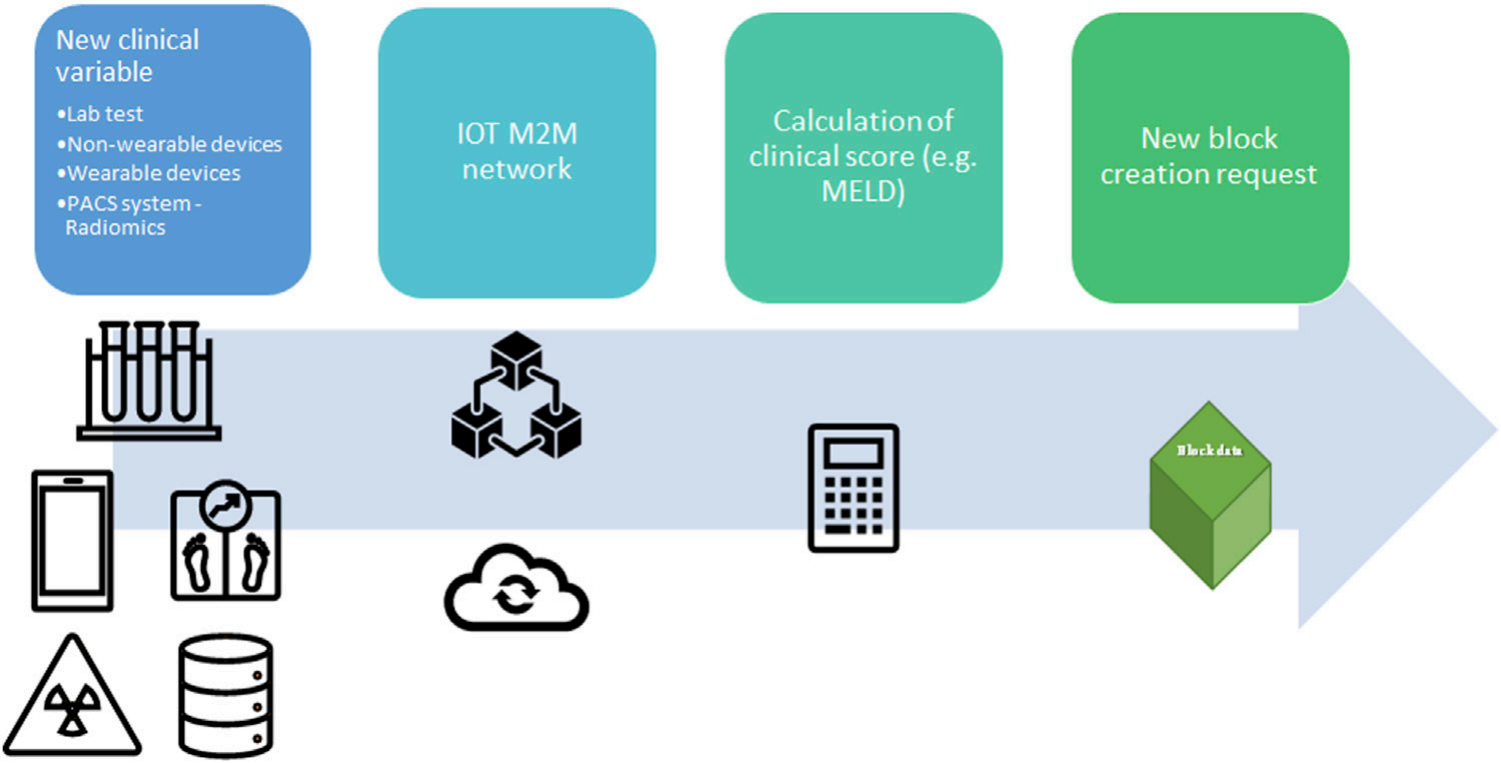
Application of Internet-of-Things (IoT) and Machine-to-machine (M2M) protocol to update the clinical value for organ allocation and patients’ waiting list. A new clinical variable is collected from wearable devices, non-wearable devices, hospital laboratory, Picture archiving and communication system (PACS), or any other facility in the hospital. The new data is transmitted in the local network between two different nodes (machine) without human interaction (M2M communication). Prior a new block request the clinical variable is integrated in clinical score (e.g., MELD score). Lab, laboratory; PACS, Picture archiving and communication system; IOT, Internet-of-Things; M2M, Machine to Machine; MELD, Model for End stage Liver Disease.

Due to the nature of the DLT, this model could determine other advantages in the organ procurement process in terms of auditability, which is immutable and can be easily reviewed by government auditors (22). However, while some authors may argue that the immutability of DLT could represent a limitation of this system in case of data entry errors, eventual data entry error may be correct by regulatory authority in private/permissioned blockchain (15). Moreover, Data entry error may be easily reduced by application of Internet of thing (IoT) technology, and machine-to-machine communication (M2M) (21). IoT is defined as a network of physical things linked to each other by means of the Internet (32), while M2M is a particular system where machines communicate without human involvement, avoiding human manipulation and securing organ allocation system (21). In fact, some of the above-mentioned organ allocation systems require biochemical variables such as PRA, INR, and bilirubin level, which can be updated directly from the laboratory in the distributor ledger, while clinical variables could be updated by medical wearable devices (21, 33), or radiological or radiomics variables could be directly uploaded from Picture archiving and communication system (PACS) (Figure 5). Finally, another potentiality of DLT decentralized nature is the lack of TTP institution for its legitimacy, leading to a real international DLT-based organ procurement network, not restricted to national borders (22). Table 3 describes the pros and cons of different systems to manage organ allocation in a centralized network, public DLT, and private/permissioned DLT (23).

**TABLE 3:** Different characteristics among Centralized, public DLT, and Permissioned DLT.

System	Centralized network	Public DLT	Permissioned/Private DLT
Costly	Yes	No	No
Ease of use	No	Yes	Yes
Speed	Current standard	Moderate	Faster than public DLT
Scalability	No	Yes	Yes
Security	Current standard	Less than current standard	More than current standard
Reliability	Yes	No	Yes
Permanent	No	Yes, DLT cannot be modified in case of data error entry.	Yes. In some cases, the owner could modify in case of data error entry.
Transparency	No	Yes	Yes
Accessibility	No	Yes	Only who is authorized by the owner (e.g., local authority) of the DLT could join.

DLT, distribution ledger technology.

Limitations of the current application are social perceptions about DLT in the medical fields, the possible conflict with European legislation, and the lack of standardization of EMR among different facilities (34). In fact, despite the promising application of DLT technology in the Estonian NHS, application of DLT in EMR is limited and scares, therefore further evidence are needed. Regarding the latter limitation, EMR standardization among different facilities could promote some benefits in terms of sharing information between different centers, and enhancing medical information migration between different providers besides transplantation (22). Finally, European privacy legislation may represent a limitation for the implementation of DLT technology in medical information technology. The General Data Protection Regulation (GDPR) is a European Union law applied from May 2018 to safeguard personal data, and privacy of European citizens (35). GDPR regulation, which was written when DLT were mostly used in cryptocurrencies and their further application were not applied in medical or other uses, introduces the “right to be forgotten” (13). In order to solve this dilemma several authors and companies are currently working to solve this paradox, with different solutions from a legal agreement between participants in a private/permissioned blockchain or improving anonymization of the data in the DLT (13).

### Expanding Living Donor Pool Through the Application of Blockchain on Crossover Programs

Despite the application of expanded criteria, deceased donors or marginal kidneys in dual kidney procedures (36, 37), it has been calculated that fewer than 25% of the waitlisted patients are transplanted because of organ shortage (38). To overcome the chronic organ shortage, kidney donation is routinely performed in one-third of patients from living donors. Moreover, besides increasing the donor pool, kidney donation from living donor determines better recipient and graft survival (39).

**FIGURE 6:**
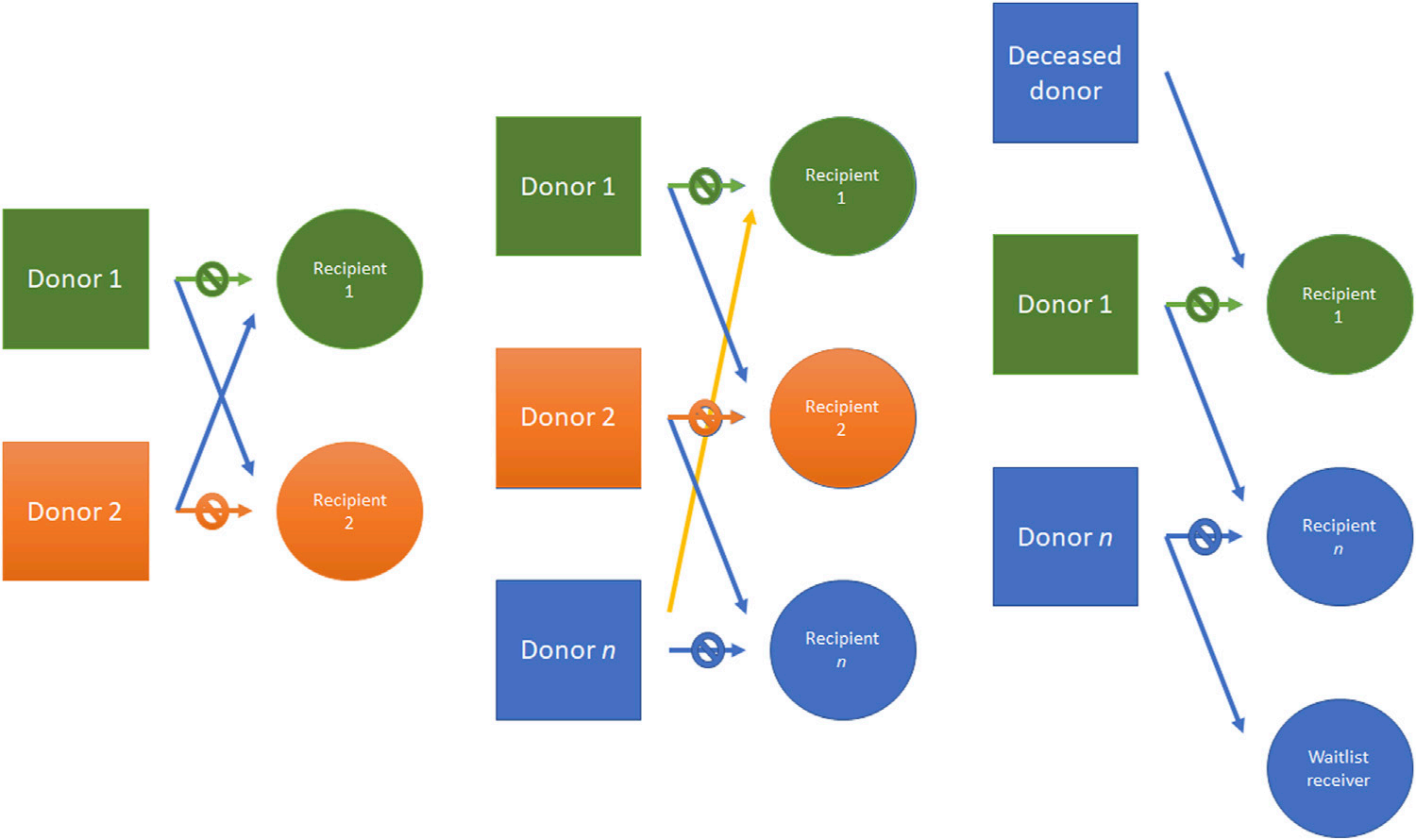
Kidney donor paired (KDP) program example. From left to right: an incompatible pair is matched with another pair; three different incompatible pairs are matched; another example is made with a deceased donor which can initiate chain donation, donating to a transplant cluster. The donation cluster may end at another donation cluster or at the deceased donor waiting list, with the end of the donor chain. KDP, Kidney donor paired.

Despite these benefits, up to 30% of patients with a possible willing living donor are not compatible with their donor, due to blood type incompatibility and previous sensitization against donor HLA (39). To overcome these limitations, different strategies have been developed as kidney paired donation (KPD). In KPD programs, an incompatible donor-recipient pair is matched with one or more pairs. In the simplest form, two incompatible pairs are matched to each other. More complex transplant chains involve a deceased transplant donor which can initiate a donation cluster. The organ is returned to the deceased donor waiting list patient at the end of the donation cluster (Figure 6). In this scenario, larger a database is needed to identify multiple pairs to increase potential transplant leading to a logistically challenging organization.

Under these circumstances, DLT could represent a great opportunity to create an international waitlist database to increase the chance of a KPD and to activate kidney donor chain (2). DLT decentralization could guarantee transparency, trustworthiness, and auditability by any node of the network (21).

### Supply Chain

#### Drugs

Immunosuppressive therapy after allograft solid organ transplantation is required to prevent rejection and preserve organ function (40–43). Various combinations of currently approved agents are needed to obtain the patients’ tailored regimens to balance adequate immunosuppression with drugs’ side effects (44–47) through continuous titration to reduce their side effects due to their narrow therapeutic index (48–51). In this light, a reliable drug supply chain is even more urgent due to the risk of counterfeit medications.

Counterfeit medications represent a major public health concern that severely impacts human lives and treatment outcomes besides transplantation. It has been calculated that, one out of ten medicines in developed countries and 1%–2% of all the drugs consumed in developed nations are counterfeit. The World Health Organization (WHO) includes products in counterfeit medications if those products are deliberately and fraudulently mislabeled with respect to source and/or identity with a difference in the package, without active ingredients, with different declared dosage, with toxic excipients or contaminants, and if active ingredients are not declared on the label or not authorized (52, 53). These factors could easily determine toxic and irreversible effects on the body and reduced graft survival.

A pharmaceutical supply chain comprehends several nodes in an end-to-end process arising from the active medication ingredients through manufacturing and delivery to patients (48, 54). As for other applications, DLT could represent a valuable opportunity to design a shared, permissioned, trusted, and decentralized platform that guarantees security, privacy, accessibility, transparency, and scalability for supply chain stakeholders (53). DLT application in drugs supply chain traceability could determine real-time tracking, improve inventory management, minimize courier costs, identify issues faster along the supply chain, and reduce errors (15).

#### Organ Transport

**FIGURE 7:**
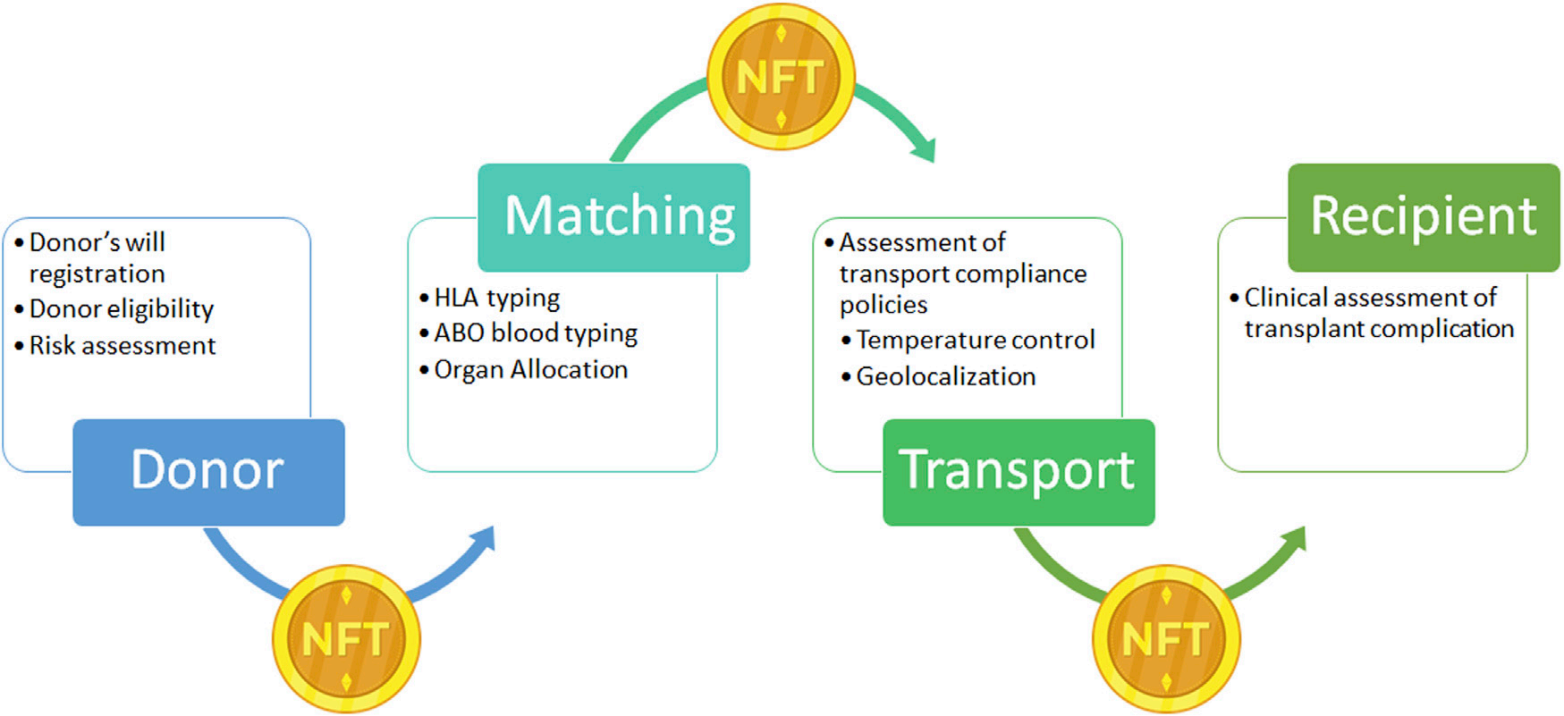
Example of Non-fungible Token (NFT) application on donor organ supply chain. NFT: is a unique digital identifier that cannot be copied, substituted, or subdivided, that is recorded in a blockchain, and that is used to certify authenticity and ownership. In the present case NFT ownership is tracked to record the different phases of the supply chain from the donor to the recipients. After registration of the donor’s will, the donor’s clinical information is registered and NFT is generated and linked to the organ donor. During the transport NFT ownership is transferred through the supply chain with the organ, to obtain a real time tracking. NFT, Non-fungible Token.

As for the drugs supply chain, donated organs could be considered a unique, high-value item. Moreover, organs as for blood products or other medical products, require stringent transport characteristics such as transport time, temperature for transport, and regulatory transport compliance policies which can be tracked in a DLT (15, 54). A proposal to apply DLT to the organ supply chain is the link of the organ with a Non-fungible Token (NFT). NFT is a non-replicable token that relies on a DLT to prove its unicity and authenticity, enabling the chance to obtain a real-time auditability and trackability of organ donor in the path from the donor to the recipient (Figure 7) as theorized for blood transplant by Booth et al. (55).

## Conclusion

In the XX century, transplantation arose as a stimulating and innovative medical field, which required an enormous effort in various medical disciplines (immunology, infectious disease, genetics, molecular biology, surgical technology, intensive care, etc.). Improvements in transplant outcomes have brought about numerous clinical and ethical dilemmas, and their solutions allowed development in medical knowledge even beyond the transplantation field (56). It is, therefore an ethical duty of the transplant community to continue to embrace innovation and overcome the limits of current systems in every medical aspect.

Currently, medical digitalization is a reality that requires all transplant personnel to play a leading role. Among the several innovations that Information and Communication Technologies could bring to transplant clinical practice, DLT could soon become of pivotal importance in overcoming some limitations of transplant programs. DLT technology, thanks to its security and scalability, could boost transplants’ programs and the reduce black market, allowing a real integration between different national health systems with real-time auditability, thanks to its distributed, efficient, secure, trackable, and immutable nature.

It is safe to assume that government-backed institutions could be extremely prudent regarding an innovation such as DLT. A supranational-based initiative by transplant physicians is needed to raise attention to the several innovations DLT could bring into transplant programs, with dedicated study groups to unveil the DLT pandora’s box. It has been calculated that 55% of healthcare applications will have adopted DLT for commercial deployment by 2025 (13). Being competitive in the future will not only be a matter of keeping pace with clinical and translational research, but also a matter of becoming leaders of technological advancement. The transplant community should thrive to get involved in the action; hence, we believe that we should not only familiarize ourselves with DLT but also look for alternative solutions to data management to drive the innovation that DLT can offer.

## Abbreviations

COVID-19, Coronavirus Disease 2019; DLT, distribution ledger technology; EMR, electronic medical records; GDPR, general data protection regulation; HLA, human leukocyte antigen; INR, international normalized ratio; IoT, internet of things; KPD, kidney paired donation; M2M, machine-to-machine communication; MELD, model for end-stage liver disease; NFT, non-fungible tokens; NHS, national health system; PACS, picture archiving and communication system; PoW, proof-of-work; PRA, panel reactive antibody; PRISMA, preferred reporting items for systematic reviews and meta-analyses; TTP, trustable third party.
